# The Potential Effect on the Number of Computerized Tomography Pulmonary Angiography if a Clinical Probability-Adjusted D-dimer Is Added to an Age-Adjusted D-dimer

**DOI:** 10.7759/cureus.80472

**Published:** 2025-03-12

**Authors:** Abin Thomas, Ritvik Sajan, Bharath Prasad S, Gireesh Kumar, Sreekrishnan Trikkur, Manna M Theresa, Sabarish Nair, Naveen Mohan, Amalendu Vijay

**Affiliations:** 1 Department of Emergency Medicine, Amrita Institute of Medical Sciences, Kochi, IND; 2 Department of Pharmacy Practice, Amrita Institute of Medical Sciences, Kochi, IND

**Keywords:** age-adjusted d-dimer, ct pulmonary angiogram (ctpa), d-dimer levels, pulmonary embolism (pe), updated diagnostic criteria

## Abstract

Introduction

Computerized tomography pulmonary angiography (CTPA) is the gold standard test for diagnosing pulmonary embolism (PE); however, owing to its high cost, contrast dye toxicity, and radiation exposure, it is necessary to ensure that CTPA is not performed unnecessarily. European Society of Cardiology (ESC) guidelines recommend the use of pretest clinical assessment tools and D-dimer levels to stratify patients according to risk, thereby determining the need for CTPA. However, such a broad classification incorporates a large population, where cases of CTPA could have been avoided. In this retrospective observational study, we established a new cohort within the moderate risk group, using a D-dimer threshold of 1000 ng/dL, and thereby aimed to identify its impact in identifying the number of cases of CTPA and to identify the number of cases of PE missed.

Materials and methods

This observational retrospective study was conducted over 24 months in the emergency medicine department of a tertiary care hospital in south India. Our study comprised of evaluation of electronic medical records of patients based on the inclusion and exclusion criteria. An overall 182 samples (n=182) were recruited, past medical records were evaluated, and classified patients were classified according to the new stratification criteria based on the Wells score, pulmonary embolism rule-out criteria (PERC), and D-dimer levels. The study outcomes of the number of CTPA cases avoided and PE cases missed out were analyzed.

Results

Assuming all samples (n=182) fell into the low moderate risk (LMR) category (Wells score<2 and PERC positive, or Wells score 2-4), 95 cases (n=95, 95/182) had D-dimer<1000 ng/dL, among which PE was absent in 93 patients (n=93, 97.89%) and present in 2 patients (n=2, 2.10%). The remaining 87 (n=87, 87/182) had D-dimer≥1000 ng/dL where all cases had PE (n=87, 100%). Assuming the samples (n=182) fell into the high moderate risk (HMR) category (Wells score 4.5-6), 21 cases (n=21, 21/182) had D-dimer< age-adjusted D-dimer (AADD) among which, PE was absent in 20 patients (n=20, 95.23 %) and present in 1 patient (n=1, 4.76%). The remaining 161 (n=161, 161/182) had D-dimer≥AADD, whereas all cases had PE (n=161, 100%). The false negative rates were 2.1% in LMR and 4.8% in HMR, while the reduction in CTPA procedures amounted to 95 scans in LMR and 21 scans in HMR.

Conclusions

We identified that establishing a higher threshold of D-dimer (1000 ng/dL) was effective in determining the need for CTPA and potentially reducing the number of CTPAs performed in suspected cases of PE.

## Introduction

Pulmonary embolism (PE) is a life-threatening condition that poses a diagnostic conundrum owing to its vague and non-specific clinical presentations (pleuritic chest pain, dyspnoea, palpitations, tachycardia, and syncope), which overlap with multiple other conditions [1-3]. Hence, imaging techniques, such as computerized tomography pulmonary angiography (CTPA), are considered to be of gold standard in PE diagnosis [4]. CTPA has a high diagnostic accuracy in detecting PE and requires only a few minutes compared to other tests [4,5]. Chooi et al. reported that CTPA has a sensitivity between 96% and 100% and a specificity between 89% and 98% in predicting PE [5].

Despite its excellent diagnostic accuracy, the decision to perform CTPA depends on different clinical prediction/probability tools such as the Wells score, pulmonary embolism rule-out criteria (PERC), Geneva score, and laboratory investigations such as the D-dimer levels [6,7]. According to European Society of Cardiology (ESC) guidelines for PE diagnosis, class 1 and level A evidence exist regarding clinical prediction/probability tools for stratifying patients into different categories based on risk for PE [6]. Multiple works of literature recommend the practice of combining D-dimer levels with clinical prediction tools to determine the need for performing CTPA [6,8-12]. According to Kearon et al., assessment using clinical pretest probability tools (Wells score) along with D-dimer is essential in risk stratification of suspected cases of PE and to identify the need to perform CTPA [8]. Westafer et al. state that using clinical prediction tools (Wells score and PERC) with D-dimer provides a higher specificity in ruling out the need for CTPA [13]. The general practice for PE diagnosis incorporates both clinical prediction tools and D-dimer levels to assess and determine the need for CTPA.

According to the ADJUST-PE study, the combination of pretest clinical probability assessment with an age-adjusted D-dimer (AADD) cutoff had a higher accuracy in ruling out PE [14]. The systematic review by Patel et al. states that D-dimer has a sensitivity of 0.97, and AADD has a higher sensitivity of 0.99 in diagnosing PE [15]. Hence, it is recommended to use a pretest clinical probability assessment tool with AADD to rule out PE risk and to determine the need for CTPA [6,14]. AADD is calculated as age × 10 ng/mL for patients aged >50 years [16].

The current practice for determining the need to perform CTPA in suspected cases of PE is based on the results of Wells score, PERC, and D-dimer levels. The patients are stratified into three risk-based categories: low risk, moderate risk, and high risk. If patients have a Wells score<2 and a negative PERC score, the D-dimer test can be avoided, and such patients will be categorized as low risk category. The moderate risk category comprises of patients with a Wells score<2 and a positive PERC or Wells score between 2-6. Among the moderate risk category, if AADD is greater than D-dimer, CTPA is avoided, while if the patient has AADD lesser than that of D-dimer, CTPA needs to be done. Patients with a Wells score>6 are classified as high risk category, and D-dimer is not necessary and can be omitted among these patients [6].

However, studies have identified that, by using a higher threshold level of D-dimer, the PE risk categorization can be improved. According to the findings of the PEGeD and YEARS study, the traditional D-dimer threshold of 500 ng/dL can be safely increased to a higher threshold of 1000 ng/dL to rule out the need for CTPA. By establishing a higher threshold for D-dimer, the sensitivity of PE diagnosis was enhanced, and the number of cases of PE missed out was reduced. This can enable in reduction of the unwanted number of CTPAs performe,d and the side effects associated with CTPA can also be reduced [17]. Based on this, we proposed to set a higher threshold for D-dimer, i.e., of<1000 ng/dL, and further subdivided the moderate risk category into low moderate risk, and high moderate risk categories according to the pretest clinical assessment tools, D-dimer, and AADD levels. We also prospectively analyzed previous patient records to identify the number of cases of PE that would be missed out according to the new proposed threshold for classification.

This retrospective observational study aimed to validate the effectiveness of establishing a higher threshold of D-dimer<1000 ng/dL, with respect to the traditional AADD as a primary objective. Furthermore, by establishing new cohorts of low moderate risk (LMR) and high moderate risk (HMR) within the moderate risk category, we aimed to identify the number of cases where CTPA could have been avoided in the emergency medicine department of a tertiary care center, which predominantly comprises the Indian population.

## Materials and methods

Study design and setting

This is a retrospective observational single-centered study conducted over 24 months (2021-2023) in the emergency department of a south Indian tertiary care hospital. The institutional review board, the ethics committee of Amrita School of Medical Sciences, Kochi, approved the clearance for conducting the study, stating no ethical concerns being raised against or violated. As the study was retrospective in nature and involved only the Electronic Medical Records (EMR) of the patients, informed consent was waived, which was approved by the institutional review board. All previous EMR data of the patients over the past 24 months were analyzed.

Study participants

**Figure 1:**
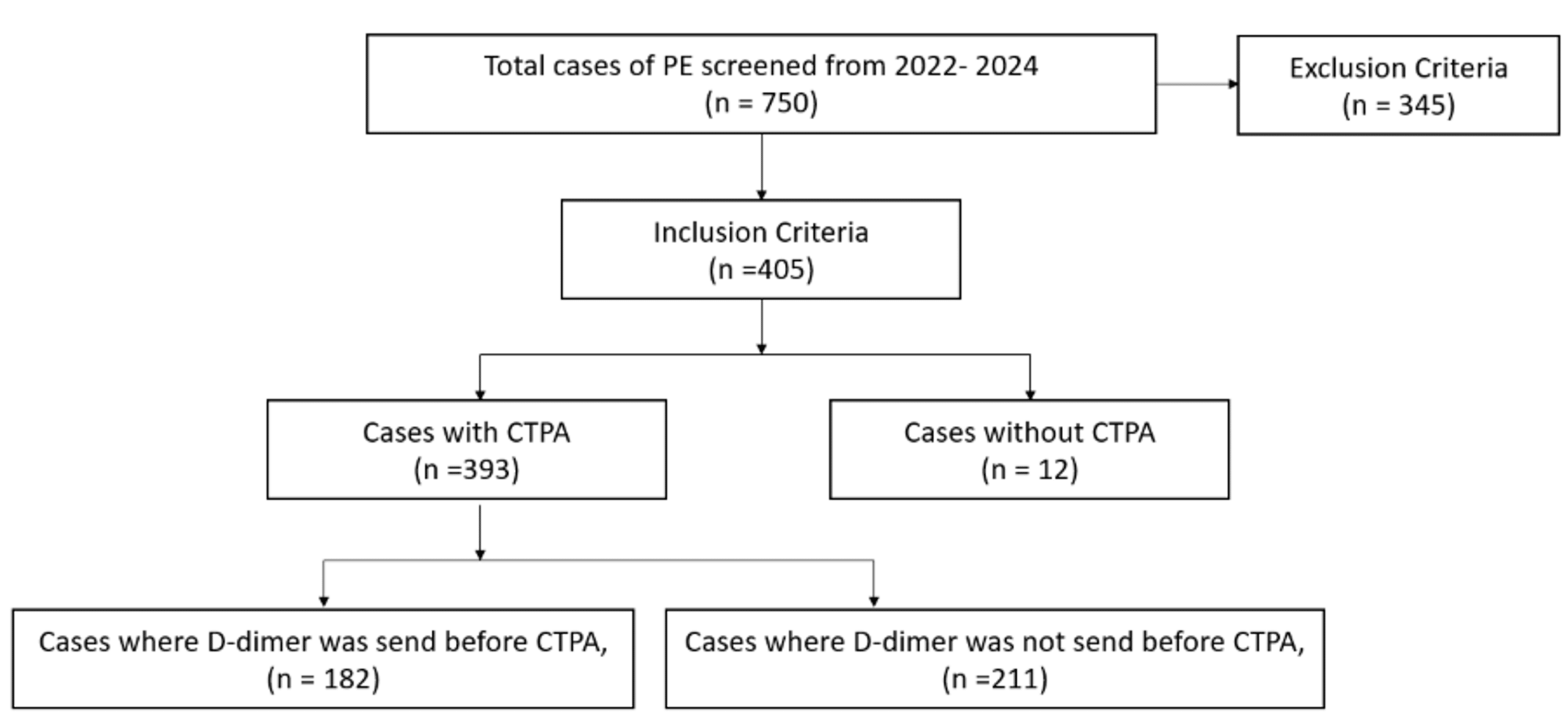
Participant screening. CTPA - Computerized tomography pulmonary angiography.

The study population was screened according to the inclusion and exclusion criteria of the study. Inclusion criteria comprised of medical records of all adult patients (age ≥18 years) presented in the emergency medicine department for whom D-dimer level and CTPA were performed, suspecting PE. Exclusion criteria comprised of patients with conditions that cause elevated D-dimer levels (cancer, deep vein thrombosis, stroke, snakebite, COVID-19, and pregnancy), individuals who expired before the initial D-dimer could be recorded, and individuals who left the hospital against medical advice before completion of data collection. Among a total of 750 patients screened, 405 patients were included based on the inclusion and exclusion criteria, and 357 were excluded. Among 405 included, 393 had CTPA performed while others had died, were discharged against medical advice, or were not consenting to treatment. Out of the 393 samples, 182 (n=182, 46.31%) had D-dimer done prior to performing CTPA, while 211 (n=211, 53.68%) did not. Hence, 182 prospective data records of patients were analyzed using the institution's EMR system. The sample screening process has been summarised in Figure 1 below. Based on previous studies, we identified 182 patients that would be sufficient for the objectives of our study.

Objectives

Our study primarily aimed to validate the effectiveness and potential impact in the number of CTPAs if a clinical probability adjusted D-dimer, of 1000 ng/dL, is added to an AADD, which was analyzed using the data obtained from previous EMR of the study participants. We also analyzed the number of missed PE cases that would have occurred if these changes had been adopted.

Methodology

Based on the inclusion and exclusion criteria, the admission demographic details of all participants were analyzed using EMR. Based on admission demographic details, the Wells score and PERC score were calculated if all necessary parameters were available to calculate them. If there is a lack of data regarding the availability of exact admission Wells and PERC scores for performing CTPA, then the patients would be assumed to be of both the LMR category and the HMR category. The LMR category should comprise patients with a Wells score<2 and PERC positive or Wells score between 2 and 4, while the HMR category should comprise patients with a Wells score between 4.5 and 6. In the absence of necessary parameters for identification of admission Wells and PERC or if the exact Wells and PERC score which led to the decision of performing CTPA is unavailable in EMR, then all the samples would be assumed to be of LMR and HMR respectively, and the study objectives will be analyzed in both groups. Further D-dimer levels and study objectives will be analyzed in all samples.

**Figure 2:**
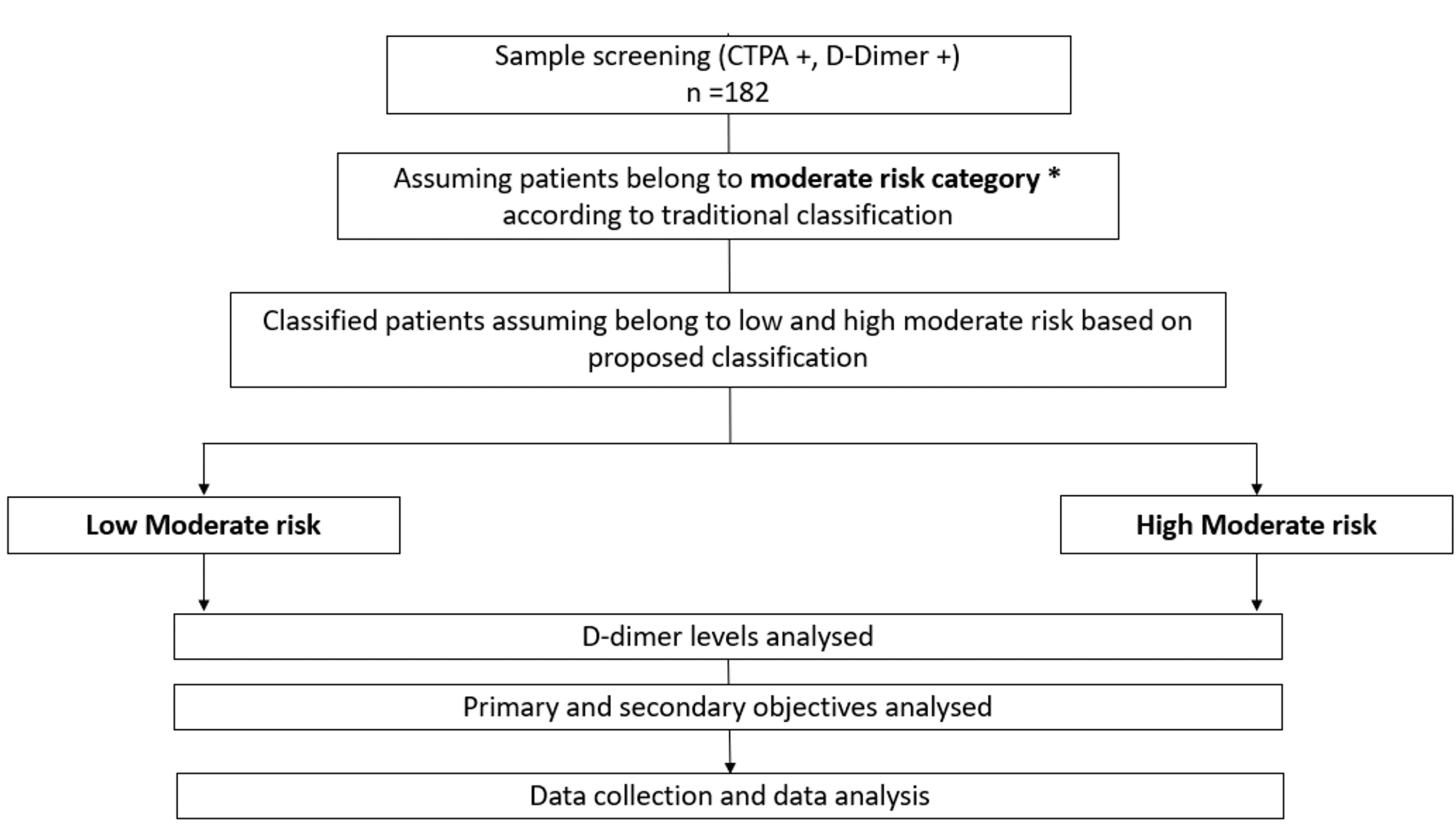
Representation of the study methodology. * Patients with Wells score<2 and a positive PERC or Wells score between 2–6, CTPA: computerized tomography pulmonary angiography.

Our study hypothesis was that in samples within the LMR category with a D-dimer level less than 1000 ng/dL, the need for CTPA can be avoided, and if greater than 1000 ng/dL, CTPA is necessary. In the HMR category, if the D-dimer is less than AADD, CTPA can be avoided, and if the D-dimer is≥AADD, CTPA has to be done. The past EMR of the patients was analysed, and study objectives were measured. The study methodology has been summarized in Figure 2 below.

Statistical analysis

Based on the proportion of reduction in CTPA in the previous studies, estimating a single proportion (relative precision) and based on assumptions that the outcome variable measure should be binary (success/failure, alive/dead), p is the probability of success in each trial; (1−p) is the probability of failure and the sampling distribution of the sample proportion (p) is approximated to normal. Using the formula;

$n = \frac{(Z^2_{1-\frac{\alpha}{2}}) \times (1 - p) \times p}{\xi^2 \times p}$

where p is the expected proportion, ξ is the relative precision, and 1−α/2 is the desired confidence level, the minimum sample size is 182. All statistical analyses were conducted using IBM SPSS Statistics version 20.

## Results

Over 24 months, a total of 393 CTPAs were performed, with 182 (n=182, 46.31%) preceded by a D-dimer test and 211 (n=211, 53.68%) without it. Based on the history of D-dimer performed before CTPA, the total sample included in our study was 182 (n=182, 100%). This is represented in Table 1 below.

**Table 1:** Number of CTPAs based on D-Dimer availability. CTPA: computerized tomography pulmonary angiography.

Category	Number of CTPAs	Percentage
With D-Dimer test	182	46.3%
Without D-Dimer test	211	53.7%
Total	393	100%

**Figure 3:**
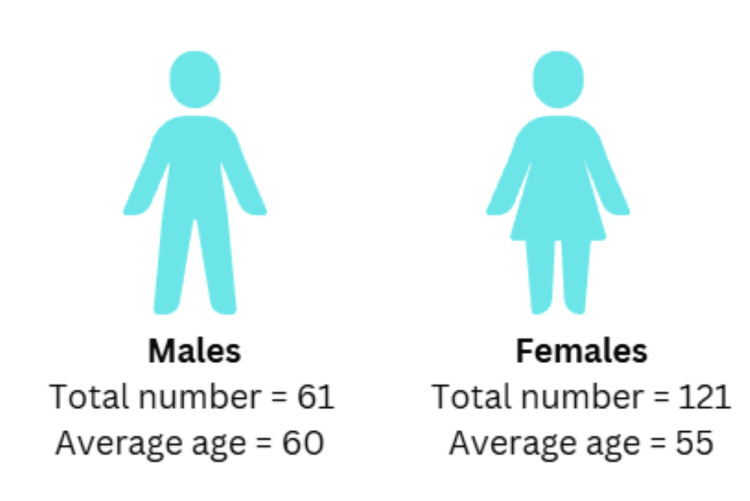
Gender and age-based distribution of participants

Among the total samples included in the study, based on gender distribution, the population was comprised of more females (n=121, 66.48%) than males (n=61, 33.52%), as shown in Figure 3.

Assuming low moderate risk category

**Figure 4:**
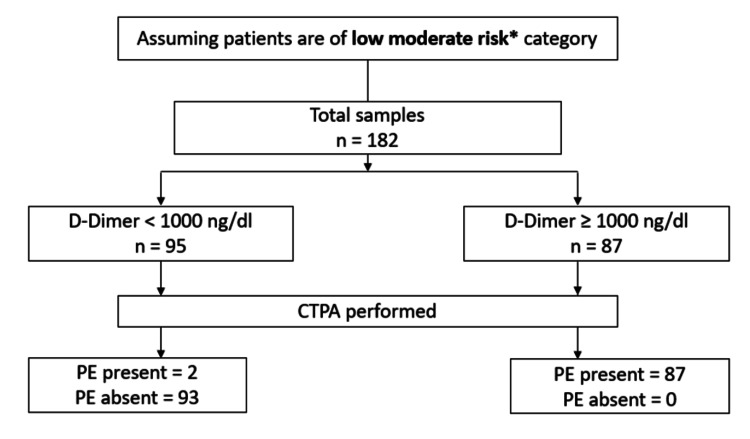
Low moderate category classification and results. * Wells<2 and PERC positive, or Wells 2-4, PE: pulmonary embolism.

If we assume that all patients (n=182) belong to the LMR category (Wells<2 and PERC positive, or Wells 2-4; see Figure 6), and if the proposed probability adjusted D-dimer threshold of 1000 ng/dl is applied, then 95 cases (95/182) had D-dimer levels<1000 ng/dl thereby requiring no need to perform CTPA. On the other hand, 87 cases (87/182) had D-dimer≥1000 ng/dL, thereby requiring CTPA. The results are represented in Figure 4 below.

Among the 95 patients (n=95) for whom CTPA could be avoided based on our hypothesis, two patients (n=2) were positive for PE, and 93 patients (n=93) were negative for PE. Hence, there could be potentially two (2/95, 2.1%) false negative results. Hence, the number of cases of PE that were missed if the probability-adjusted D-dimer (<1000 ng/dL) was adopted was two cases.

Assuming high moderate risk category

**Figure 5:**
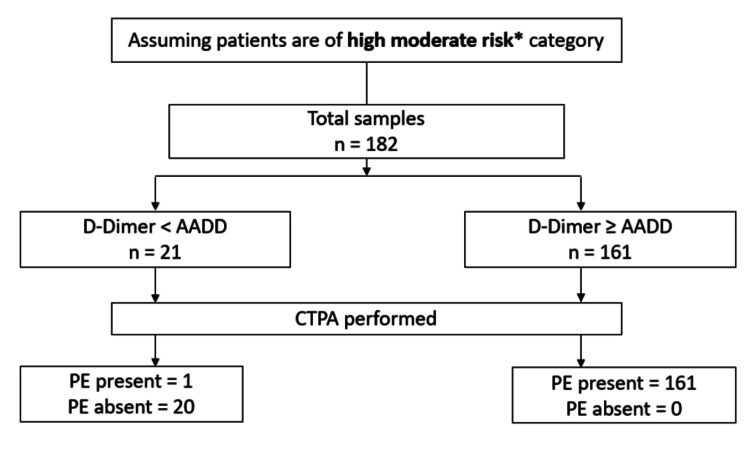
High moderate risk category classification and results. * Wells score 4.5–6, AADD: age adjusted D-dimer, PE: pulmonary embolism.

If we assume all patients (n=182) were classified as high moderate risk (Wells score 4.5-6), and the AADD threshold is applied, then 21 samples (21/182) would have D-dimer less than AADD, thereby requiring no need to perform CTPA. However, 161 samples (161/182) would have D-dimer more than AADD, requiring CTPA. The results are depicted in Figure 5 below.

Among the 21 cases (n=21) for whom CTPA could be avoided, one case was positive for PE (1/21, 4.8%), thereby being the potential false negative result. Hence, the number of cases of PE which was missed if the AADD (D-dimer<AADD) was adopted was one case.

In the LMR category, among the total 182 cases, in the cohort where D-dimer<1000 ng/dL, 2 cases (n=2, 2.10 %) showed PE by CTPA, while the remaining 93 cases (n=93, 97.89%) were free of PE. In the cohort where D-dimer≥1000 ng/dL, 87 cases (n=87, 100%) had PE. So, there is a statistically significant difference in the findings of PE by CTPA in the LMR category (p<0.001).

In the HMR category, among the total 182 cases, the cohort where D-dimer<AADD, 1 case (n=1, 4.76 %) showed PE by CTPA, while the remaining 20 cases (n=20, 95.23 %) were free of PE. In the cohort where D-dimer≥AADD, 161 cases (n=161, 100%) had PE. So, it shows a statistically significant difference in the findings of PE by CTPA in the HMR category (p<0.001).

There could be a reduction in CTPA-positive procedures to 95 scans (182-87) in low moderate risk to 21 (182-161) scans in high moderate risk, with false negative D-dimer rates ranging (RS1) between 2.1% to 4.8% in LMR and HMR, respectively. The CTPA reduction rates in low moderate risk are 52.2% reduction (95/182x100=52.2%) and in high moderate risk 11.5% reduction (21/182x100=11.5%).

## Discussion

This retrospective observational study aimed to identify the impact on the number of CTPAs if a clinical probability-adjusted D-dimer is added to an AADD in patients admitted to emergency department settings. A total of 182 participants (n=182) were included in the study, and the primary and secondary outcomes were analyzed based on the data available from EMR.

**Figure 6:**
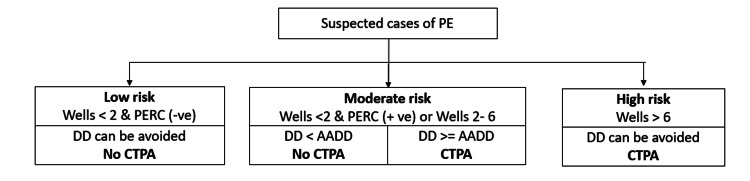
Summarised representation of guidelines followed in determining need for CTPA. Image credits: Authors PE: pulmonary embolism, PERC: pulmonary embolism rule-out criteria, DD: D-dimer, AADD: age-adjusted D-dimer, CTPA: computerized tomography pulmonary angiography.

The ESC guidelines for the diagnosis of PE and for determining the need to do CTPA have been followed due to their high diagnostic accuracy and specificity [6]. This primarily involves the stratification of patients into different risk categories based on pretest clinical probability assessment tools (Wells Score, PERC), D-dimer, and AADD levels. This is represented in Figure 6 below.

**Figure 7:**
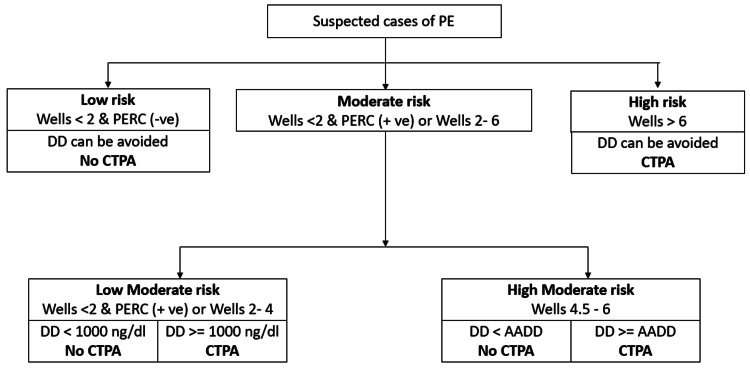
New guidelines for determining CTPA. Image credits: Authors PE: pulmonary embolism, PERC: pulmonary embolism rule-out criteria, DD: D-dimer, AADD: age-adjusted D-dimer, CTPA: computerized tomography pulmonary angiography.

Our study aimed to identify the diagnostic predictability by establishing a new cohort within the moderate risk category and using a threshold of 1000 ng/dL as a new cut-off for CTPA screening. The proposed stratification method in determining the need to perform CTPA has been represented in Figure 7 below.

Assuming patients (n=182) were of LMR, based on our proposed guidelines, among the patients with D-dimer<1000 ng/dL (n=95), PE was absent in 93 (n=93, 97.89%), and PE was present in 2 (n=2, 2.10%). Hence, the CTPA imaging reduction rate in LMR was 52.2% (n=95) with two false negatives (2/95, 2.1%). Assuming patients (n=182) were of HMR, among the patients with D-dimer< AADD (n=21), PE was absent in 20 (n=20, 95.23%), and PE was present in one (n=1, 4.77%). Hence, the CTPA imaging reduction rate in HMR was 11.5% (n=21) with one false negative (1/21, 4.8%). The imaging reduction rates in our study were different, 52.2 % in LMR and 11.5 % in HMR, respectively, while previous studies such as YEARS and PEGeD had 8.7% and 8.6%, respectively, where the reduction was calculated in comparison to an age-adjusted strategy [8,16]. This difference could be attributed to differences in our study methodology, small sample size, and difference in pretest clinical probability assessment tools.

However, in our study, deviations from the guidelines recommendations of performing CTPA are observable as all patients 182 (n=182) have had CTPA performed. This could be attributed to a lack of initial pretest clinical assessment scores, changes from initial pretest clinical assessment scores from the time of presentation leading to a need to perform CTPA, clinical deterioration of the patient, and the clinician’s judgment. Similar findings are reported in the study by Banks et al., where one-third of participants (32.4%) with D-dimer between≥500 and <1000 ng/mL had CTPA performed [17]. Multiple studies report this rising concern of the high use of CTPA instead of utilizing pretest assessment tools in diagnosis [17-19]. This highlights the necessity of awareness regarding CTPA use and the overwhelming concern of unwanted CTPA leading to radiation exposure, contrast nephropathy risk, and cost-effectiveness.

Strengths and limitations

Our study was able to identify the number of unwanted CTPAs, which can be reduced by establishing a cutoff threshold of 1000 ng/dL for ruling out the need for CTPA contrary to the traditional guidelines. We also validated the number of false negatives associated with adopting such a practice among a south Indian population. Also, we were able to identify the need for awareness regarding the use of pretest clinical assessment tools along with D-dimer to determine the need for CTPA rather than relying on hunches.

Our study limitations primarily comprised of unavailability of the exact Wells score and PERC score at admission, thereby affecting the stratification criteria, hence affecting our study methodology to assume all patients to be of a specific cohort. The retrospective nature of the study design also is arguable as a limitation; hence, we aim to perform a prospective study in the future aiming to overcome the study's limitations. The single-center nature of the study also affected the generalisability of the results, as this study focused specifically on a single tertiary care hospital catering to a single geographical location, particularly south India.

## Conclusions

This retrospective study, conducted in the emergency medicine department of a tertiary care center in South India, aimed to evaluate the effectiveness of a revised D-dimer threshold in optimizing the diagnostic approach for suspected pulmonary embolism (PE). By establishing a new D-dimer threshold of 1000 ng/dL and integrating it with pretest clinical assessment tools such as the Wells score and PERF score, the study demonstrated a more refined strategy for determining the necessity of computed tomography pulmonary angiography (CTPA). The findings suggest that this combined approach enhances diagnostic accuracy while significantly reducing the number of unnecessary CTPA procedures, thereby minimizing radiation exposure, contrast-related risks, and healthcare costs.
